# Introductory and Other Addresses

**Published:** 1851-08

**Authors:** 


*Introductory and other Addresses.*—We received, from time to time, during the last winter, Introductory and other Addresses, which we filed by themselves with the intention of devoting an article to the subject, and reviewing them severally. After so much delay, however, the expediency of of carrying this design into execution becomes questionable, were there no obstacles on the score of leisure and space. The season is near at hand when a fresh crop is to be looked for; and with not a few publications of this sort, the interest for the reader is not enhanced by time. They do not, like sound wine, improve with age, but, like waters impregnated with *gas*, are only attractive during effervescence.

We would not be thought to disparage this branch of medical literature. It embraces many interesting and valuable productions, and not a year passes without additions to the list of those which richly deserve, not only to be read, but preserved. Several addresses among those published during the past year, claim attention at the hand of the medical journalist, in behalf of their intrinsic excellence, and it is injustice to both authors and our readers, not to have given them seasonable and extended notice in our columns. Desirous of making some slight amends for not being able to fulfill our original intention, we append a list of the addresses which we have received, with the subjects, whenever the latter are enunciated by the authors:—

1. Impediments to the Study of Medicine. By J. K. Mitchell, Professor in the Jefferson Med. College, Philadelphia. Delivered Nov. 8, 1850.

2. The Obstacles opposing the Progress of Medical Science. By Horace Green, A. M., M. D., Prof. in the New York Med. College. Delivered Oct. 27, 1850.

3. An Introductory Lecture delivered at the the opening of the thirty-first session of the Medical College of Ohio, Nov. 4, 1850. By John Bell, M. D., Prof. in Med. College of Ohio.

4. A Discourse pronounced before the class of Starling Medical College, at the opening of the winter session of 1850—51. By Samuel Hanbury Smith, M. D., formerly Prof. in Starling Med. College, Superintendent of Ohio Lunatic Asylum, etc. etc.

5. An Introductory Lecture, delivered at the opening of the Kentucky School of Medicine. By Samuel Annan, M. D., Prof. in Med. Depart. of Transylvania University.

6. On Success in the Medical Profession. By John Ware, M. D., Prof. in Mass. Med. College.

7. A Historical Sketch of Surgery, from the revival of Literature to the end of the seventeenth century. By Jas. Bryan, M. D., Prof. in Geneva Med. College.

8. Progress of Medicine during the first half the nineteenth century. By James Bryan, M. D., Prof. in Philadelphia College of Medicine.

9. Charge to the Graduates of Jefferson Medical College of Philadelphia. By Prof. Thomas D. Mutter.

10. On the Comparative Intellectual Standing of the Medical Profession. By E. R. Peaslee, A. M., M. D., Prof. in Med. School of Maine.

11. The Present Tendency of Investigation in Medicine. By Samuel Parkman, M. D., M. M. S. S., one of the Surgeons of the Mass. Gen. Hospital.

